# A Much Under-Paid Profession

**Published:** 1920-03-13

**Authors:** 


*To the Editor of* THE HOSPITAL.

### A Much Under-paid Profession.

*To the Editor of* THE HOSPITAL.

SIR,—I was very glad to read "Hospital Secretary's" letter in answer to mine, and to know that somebody else thinks "that nurses' salaries in many (might I add in all?) instances need reconsideration on liberal lines." I had no idea, or desire, of stating their case unfairly by omitting the well-known fact that most of them are boarded, lodged, with laundry expenses, and, in a few instances, have a uniform allowance. This is beside the point at issue. My object in writing was to emphasise the appalling fact that the Chelsea Guardians by their action in voting an increase of 9¼d. per week to the nursing staff, had totally under-estimated their worth, to say nothing of this action being an insult to the profession. What employers to-day would dare to give the smallest boy or girl in their factory less than 1s. per week rise? And yet women of intellect have been given 9¼d. per week. "Shameful." My contention is to pay fully trained sisters and nurses holding responsible positions the salary of £40 to £45 per year, plus a 30 per cent. bonus, is nothing short of a distinct injustice, and the writer sincerely hopes that this stigma on so honourable a calling will be removed.

Let me give a comparison (if I may call it such) which appeared in *The Times* for March 5, 1920. Three different situations for cooks, and in each the wages were £40 to £45, with board, lodging, laundry, and shorter hours, by a long way. Even to-day, when we hear so much talk about an 8-hour day for nurses, in one of the largest hospitals in London they come on duty at 7 A.M., and are not off till 8.30 to 9.30 P.M., having about three hours off during the days, which is an eleven-hour day! No wonder they break down in health.

It is up to the College of Nursing to bring all the influence they can to bear upon those responsible for the whole welfare of our nursing profession, and fight for a substantial all-round increase of salaries, better hours, a more liberal dietary, more time off per diem, a keener consideration when run down in health, and seeing to it that a nurse is not allowed to carry on till she literally drops.

GUARDIAN.

